# Supplementary material for: Effective breastfeeding techniques and associated factors among lactating women in Ethiopia: A systematic review and meta-analysis
Source: PLoS One. 2024 Jun 27;19(6):e0306167. doi: 10.1371/journal.pone.0306167 (PMC11210781; doi:10.1371/journal.pone.0306167)
Supplement: Attachment — Submitted filename: comment plos one.docx [file pone.0306167.s004.docx]

Dear Editorial Team and Authors: You asked me to review this study titled “Prevalence and Associated Factors of Effective Breastfeeding Techniques among Lactating Women in Ethiopia: A Systematic Review and Meta-Analysis” and I am so grateful for the opportunity. The issue is intriguing. However, it comes with a lot of limitations. I will be glad to suggest the paper's publication if the author attempts to address the following topics: Carefully check the comments I've written below.

1. Even if the title is very interesting, what is the importance of studying effective breast feeding technique rather than studying ineffective technique? In abstract section you are going to study the effective breast feeding technique but you are talking with ineffective, so there is contrary idea there.
2. There is error in punctuation marks and language.
3. In abstract section make it consistent way of writing abstract either structured and unstructured. But in the Plos one journal the is structured abstract writing format is recommended …. First, the abstract is suggested to be presented in the way of background, objectives, methods, results, conclusions, and implications.
4. Title is not consistent with what you had written in introduction. The introduction is suggested to be rewritten.
5. In result, reference should be cited appropriately
6. The tables in the results section are recommended to be presented in a three-line table. The current presentation format is very unfriendly.
7. There is copy writing or plagiarism
8. Where is the statistical measure of publication bias?
9. In discussion section are going to discuss systematic review with single study? Try to discuss systematic review finding with systematic reports, unless it is not discussed
10. Error! Reference source not found.) what is this about???
